# Effect of Soluble Glass Alkali Activation on the Geotechnical Performance of Sandy-Pebble Soil Stabilized with Biomass Bottom Ash

**DOI:** 10.3390/ma19102169

**Published:** 2026-05-21

**Authors:** Danutė Vaičiukynienė, Gediminas Stelmokaitis, Petros Christou

**Affiliations:** 1 Faculty of Architecture and Civil Engineering, Kaunas University of Technology, Studentų St. 48, 51367 Kaunas, Lithuania; gediminas.stelmokaitis@ktu.lt; 2 Department of Civil Engineering, Frederick University, 1036 Nicosia, Cyprus; petros.m.christou@gmail.com

**Keywords:** soil stabilization, alkali activated ash, N-A-S-H and C-S-H gels, geopolymerization reactions

## Abstract

The purpose of this study was to evaluate how an alkali activator, specifically soluble glass, influences the geotechnical performance of sandy-pebble soil when combined with biomass bottom ash (BMA) as a sustainable stabilizing material. This work focused on understanding whether alkali activation could increase the strength, compactness, and overall engineering suitability of these mixtures while also examining how the activator affects permeability. To accomplish this, mixtures containing different proportions of BMA were prepared and treated with soluble glass at controlled water-to-activator ratios, followed by standard geotechnical procedures including Proctor compaction and California Bearing Ratio testing to assess density and load-bearing capacity. The results showed that soluble glass substantially improved the mechanical behavior of the mixtures, with both Proctor density values varying from 1.48 to 2.04 Mg/m^3^, depending on BMA content and activator dosage, while CBR values more than doubled for mixtures containing 20% BMA at a water-to-soluble-glass ratio of 1:3. Water permeability decreased with increasing BMA and activator content, from 8.11 × 10^−5^ to 5.91 × 10^−5^ m/s, although the permeability threshold of ≤2 × 10^−5^ m/s was not reached. These enhancements were linked to better packing of soil particles due to the void-filling effect of BMA and the formation of new binding compounds produced through alkali-activation reactions, including N-A-S-H and C-S-H gels. However, this study also found that higher amounts of soluble glass reduced water permeability, an effect associated with the denser microstructure created during geopolymerization. Overall, the findings demonstrate that stabilizing sandy-pebble soil with alkali-activated BMA is an effective approach to improving essential geotechnical properties while simultaneously offering environmental benefits by repurposing biomass waste in ground-improvement applications.

## 1. Introduction

Biomass is considered to be one of the most significant sources of renewable energy globally, especially in the field of heat generation, where it accounts for 70–75% of the total biomass energy consumption [1]. The use of biomass in district and domestic heating systems has increased significantly in recent decades across the European Union, especially in Sweden, Finland, Lithuania and Estonia [2]. According to the Lithuanian Energy Agency, in 2023, Lithuania will generate around 5.5 TWh of energy from biofuels, which corresponds to 5–6 million tons of wood fuel [3]. Wood has an average ash content of 2%, which indicates that 100,000 tons of biomass ash waste are generated per year and subsequently sent to landfill [4,5]. The International Energy Agency forecasts that biomass will continue to be one of the world’s primary renewable heat sources by the year 2030 [1]. The recycling of some types of biomass ash is problematic due to the large amount of non-reactive parts such as quartz and calcium carbonate. For this reason, these ashes are not often used as supplementary cementitious materials (SCM) due to their low pozzolanic activity [6]. A similar situation exists with alkaline-activated binders, low pozzolanic activity ash, which are not suitable for use separately as precursors. In such cases, it is recommended to create mixed aluminosilicate precursors by including more reactive aluminosilicate materials [7].

There are several studies where biomass ash has been used to stabilize soils during road construction. Barišić et al. [8] used three types of biomass ash: barley, sunflower seed husks and wheat to replace lime in hydraulically stabilized soil. It was determined that the strength properties of soil–lime–fly ash mixtures were closely related to the chemical composition of ash. Iyaruk et al. [9] used biomass bottom ash and cement to stabilize lateritic soil. The best results were achieved with a mixture of 80% biomass bottom ash (maximum ash content) and 5% cement. This formulation had the best geotechnical engineering properties, such as the modified Proctor test, the California Bearing Ratio (CBR) test and the unconfined compression test, and it did not leach heavy metals. A study [10] investigated the potential of biomass bottom ash to stabilize expansive clay from highway construction. The addition of biomass bottom ash as a filler material to the expansive clay soil of transport roads improved the mechanical properties and reduced the level of expansivity. The free swelling capacity of the expansive clay was 11.64%, whereas the addition of biomass bottom ash reduced the free swelling capacity and was in the range of 6.5% and 4.9%. Jamnongwong et al. [11] demonstrates that dam sediments stabilized with a 10% eucalyptus ash–cement blend achieve substantial mechanical gains (UCS up to 2.25 MPa), exhibit denser microstructures with C–S–H formation, meet environmental safety standards through low metal leachability and compliant groundwater quality, and offer a cost-effective, sustainable alternative to conventional road construction materials. Alkaline activation of rocks can also be applied to the stabilization of mudstone-cemented soft rock during tunnel construction [12]. Recent findings by Xue and Wu [13] show that cyclic thermal treatment fundamentally transforms the mechanical behavior and brittleness evolution of geomaterials, underscoring the broader importance of understanding how external chemical or thermal actions modify fracture development and structural performance—an aspect also central to evaluating stabilization mechanisms in alkali-activated granular soils.

Recently, a lot of research has focused on the stabilization of soils using alkali-activated materials. Morales et al. [14] validated a chemical method for stabilizing lateritic gravelly soils. In this case, sugarcane bagasse ash was used as an aluminosilicates precursor to produce an alkali-activated binder. As an alkaline activator, a sodium hydroxide solution was prepared at different concentrations ranging from 3 to 10 moles. The inclusion of alkali-activated ash binder in the system led to improvement in mechanical strength (unconfined compressive strength) and durability, and compaction properties were modified. Geomaterial for use in pavement subbase was suggested. Anburuvel et al. [15] used the alkaline activation of biomass ash to stabilize lateritic soils in road structures. Alkali-activated eggshell ash and rice husk ash with a sodium hydroxide solution improved the mechanical strength of the soil and could be a potential substitute for cement stabilization of this soil. Geopolymer consisting of eggshell ash and rice husk ash improved the mechanical properties of stabilized lateritic soil. The best results were obtained with the following geopolymer compositions: 3% or 2% eggshell ash, 1% or 2% rice husk ash and 1% NaOH. Previous studies [16] have shown that fly ash and blast furnace slag geopolymer can be used to stabilize problematic dusty clay. The ratio of the aluminosilicate precursors (fly ash and blast furnace slag), the concentration of the alkali activator (sodium hydroxide solution) and the curing temperature were significant factors. The results show that unconfined compressive strength is closely related to the geopolymerization process and interparticle forces. Higher curing temperatures increased the rate of geopolymerization. The best ash-to-blast-furnace-slag ratio was 20:10. Santhikala et al. [17] prepared a geopolymer from a blended precursor made from fly ash, metallurgical slag, biomass ash and an alkaline activator to stabilize expansive soils. It was found that the unconfined compressive strength of the expansive soils to be stabilized increases with increasing geopolymer content. The optimum activator concentrations were 8 M and 10 M. It was recommended to use at least 20% geopolymer for soil stabilization. For the stabilization of soft soils, Wu et al. [18] proposed the use of a geopolymer made from ash and slag. After 28 days, the unconsolidated compressive strength was 2 MPa. The ratio of slag to fly ash was 1:1, and the concentration of alkali activator was 6% and its content 0.6. In this stabilized soil, calcium silicate hydrate and sodium aluminosilicate hydrate were formed as binding compounds which increase the density due to the filling effect and chemical reactions. Another study [19] investigated the sustainable stabilization of sandy soil using alkali-activated binders produced from construction and demolition waste, demonstrating their mechanical effectiveness, durability, and environmental advantages compared with conventional cement-based stabilization. Microstructural analysis confirmed the development of N–A–S–H and mixed aluminosilicate–calcium silicate hydrate gels. Pourakbar et al. [20] shows that treating clayey soil with 2.5% alkali-activated sewage sludge increases its strength from 176 kPa to 1.46 MPa, improves shear parameters, densifies the microstructure, and reduces metal leachability, demonstrating its potential as a low-carbon alternative to cement and lime. SEM analysis reveals that alkali-activated sewage sludge produces a denser and more cohesive soil matrix by significantly reducing pore spaces and enhancing particle bonding.

However, despite extensive research on biomass ash and alkali-activated binders for soil stabilization, no studies have examined the use of soluble glass as an activator for mixtures of sandy pebbles and biomass bottom ash, nor its influence on key geotechnical engineering properties. The interaction mechanisms between soluble glass and low-reactivity biomass ash in granular soils also remain insufficiently understood.

The novelty of this study lies in evaluating soluble glass as a chemical stabilizer for sand–biomass bottom ash mixtures and determining its optimal dosage. This approach introduces a new application pathway for low-reactivity biomass ash and provides new insights into the stabilization mechanisms of alkali-activated granular soils.

The aim of this study was to investigate the effect of soluble glass on the main geotechnical engineering properties of sand–pebble and biomass bottom ash soil mixtures and to determine the optimal amount required for effective stabilization.

## 2. Materials and Methods

### 2.1. Experimental Techniques

X-ray fluorescence (XRF) was conducted for elemental analysis of ordinary Portland cement (OPC) and biomass bottom ash (BMA). It was performed using an X-ray fluorescence spectrometer (Bruker X-ray S8 Tiger WD, Karlsruhe, Germany) using a Rh tube, an anode voltage Ua of up to 60 kV, and current I of up to 130 mA.

X-ray diffraction analysis (XRD) of BMA was performed using a D8 Advance diffractometer (Bruker AXS, Karlsruhe, Germany) operating at a tube voltage of 40 kV and tube current of 40 mA. The X-ray beam was filtered with a Ni 0.02 mm filter to select the CuKα wavelength. The powder X-ray diffraction patterns were identified with references available in the PDF-2 database.

The specific surface area of OPC was measured with the Blaine instrument according to the EN 196-6 standard [21].

The microstructure of BMA and stability of sandy-pebble soil and biomass bottom ash soil blends by using alkali activation was studied by scanning electronic microscopy (SEM) using a high-resolution scanning electron microscope FEI Quanta 200 FEG with a Schottky field emission gun (FEG).

The analysis of the prepared solutions was carried out by flame atomic absorption spectrophotometry with a SHIMADZU AA-7000 atomic absorption spectrophotometer (Shimadzu Corporation, Kyoto, Japan), using a cathode-free discharge lamp (10 mA, λ = 357.9 nm). The following conditions were used for the analysis: atomization gas: air–acetylene mixture (flow rate 2.8 L/min); flame height, 9 mm; slit width, 7 mm; calibration curve: 0; 0.2; 0.5; 1.0; 2.0 µg/L (ppm); typical sensitivity of the method at 1% absorbance, 0.07 ppm. The concentration of the specific compound A eluted from the sample is calculated according to Equation (1):

(1) $$A=\frac{C\times L}{M}$$

where:

- A—amount of chemical compound leached from the solid sample (mg/L).
- C—concentration of the compound in the eluate (mg/L).
- L—volume of solvent used for leaching (kg).
- M—mass of the dry solid sample (kg).

In order to assess the suitability of BMA for the production of an alkali-activated binder, it is necessary to assess the pozzolanic activity of the ash in terms of the strength activity index (SAI), which is evaluated according to EN 450-1 [22]. Thus, the SAI was determined after 7 and 28 days by averaging the compressive strength of three samples using Equation (2):

(2) $$SAI=\frac{P}{C}\times 100$$

where:

- P—average compressive strength of mortar containing BMA (MPa).
- C—average compressive strength of reference mortar without BMA (MPa).

The compressive strength of hardened samples was evaluated after seven and twenty-eight days. The load frame of a Toni Technik 2020 (Toni Technik Baustoffprüfsysteme GmbH, Berlin, Germany) was used to compress the sample cubes. The compressive strength test was carried out in accordance with EN 13286-41 [23].

The granulometric composition is the size distribution of aggregate particles, which is an essential parameter for assessing the suitability of a material for a specific construction purpose. To accurately assess the composition of sand, gravel, and ash fractions, a standardized sieving method is applied, which is regulated by the European standard EN 933-1 [24].

Standard Proctor compaction tests of sandy-pebble and ash mixtures were performed according to EN 13286-2 [25]. The dry sandy soils were initially mixed with different proportions of ash (10%; 20%; 40%; 60%; 80%), and the Proctor density and optimal moisture content were determined. The mixtures were prepared by homogenizing dry sandy pebbles and biomass ash, followed by the addition of the required amount of water. Prior to compaction according to EN 13286-2, the moist mixture was conditioned in a sealed container for 1 h to ensure uniform moisture distribution. Initial mixing was carried out with hands.

**Figure 1:**
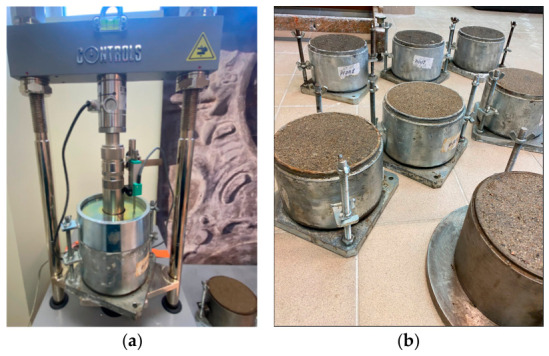
The process of CBR testing (**a**); prepared mixtures of sandy pebbles and biomass bottom ash for CBR testing (**b**).

The strength of the soil was evaluated by the California Bearing Ratio (CBR) index calculated at an embossing depth of 2.5 mm or 5.0 mm. The scheme of CBR testing is shown in Figure 1a.

The results of the CBR index were given as a percentage, which indicates the percentage conformity of the soil being tested with the value of the standard soil, which is equivalent to 100%. The test was carried out in accordance with the requirements of EN 13286-47 [26].

For each soil type, 3 samples were produced with a density of 100% of the Proctor density, and the result is given as the arithmetic mean of these values (Figure 1b). Individual values of the CBR for each sample were determined and computed, as well as the mean value for each mixture tested with the required additive content. The soil was mixed with biomass ash in an appropriate proportion, and then a sodium silicate solution was added to the water at an appropriate percentage at the optimum water content as determined by the Proctor test. The mixture was then placed in a cylindrical (Proctor) mold 150.0 ± 1.0 mm in diameter and 120.0 ± 1.0 mm high and compacted by means of a plough in accordance with the requirements of EN 13286-2 [25]. After curing for 7 and 28 days at 20 ± 2 °C at 95% humidity, the CBR test was carried out using a compression device with a piston force of 40 N on the samples.

The water permeability of coarse-grained and multi-grained soils and minerals was determined using the constant compression water permeability method according to EN ISO 17892-11 and is defined by the value of the water permeability coefficient [27]. The density of samples was determined according to EN 12390–7 [28].

FT-IR spectra were recorded with a Perkin Elmer FT-IR System spectrometer. A total of 1 mg of the substance was mixed with 200 mg of KBr and compressed in a forming press under vacuum for the IR analysis.

### 2.2. Initial Materials

#### 2.2.1. Main Properties of Initial Materials

In this study, biomass ash was used as a substitute for natural sandy-pebble soil mixtures in the soil stabilization blends, which is one of the main components of the modified soil mix. This ash is produced by burning biomass such as wood chips or other plant residues. The chemical and mineral composition of biomass ash is one of the main factors determining its properties and its potential impact on the performance of the modified soil system mix. Therefore, knowledge of the chemical and mineral composition of biomass ash is essential to properly understand and predict how it will perform in the cementitious system mix.

Additionally, an ordinary Portland cement of grade CEM I 42.5 R, satisfying the EN 197-1:2011 standard [29], was used for the evaluation of the biomass ash strength activity index.

The chemical compositions of the initial materials, such as OPC and BMA, are presented in Table 1.

OPC was found to be dominated by calcium oxide and silicon dioxide. The same oxides (CaO and SiO_2_) also make up the majority of BMA’s oxide composition, and the sum of these oxides is 85.9%. Portland cement had a specific surface area of 350 m^2^/kg (Blaine), a normal paste consistency of 28.5%, and compressive strength after 2/28 days of 32.3/63.1 MPa.

Atomic absorption spectrophotometry was used for the determination of the leaching of heavy metals from the biomass ash (Table 2).

In order to use biomass ash to replace part of the natural sand, for producing alkali-activated mixtures for soil stabilization, leaching tests are necessary to determine which heavy metals may be released into the environment during ash disposal. The test results show that the heavy metals vanadium (V) and lead (Pb) have the highest amount of leaching, and the amount for other metals leaching from ash is significantly lower (Table 2). The results of the leaching test on the BMA show that the content of all elements in the test solution is less than 1 mg/L, which is within the limits allowed by LST EN 12457-4:2003 [30].

The mineral composition of the biomass ash by XRD analysis shows that one of the main components of biomass ash is quartz (Figure 1a). Calcium oxide, calcium carbonate and calcium silicate hydrate were also found in the ash. Similar mineral composition of Lithuanian biomass ash was detected by Kaminskas et al. [31]. The microstructure of BMA is shown in Figure 1b.

Sodium silicate solution (Na_2_SiO_3_) with a silicate modulus of 3.0 and a density of 1290 kg/m^3^ was used as the alkali activator to improve the strength properties of the sandy-pebble soil (0/32 mm) and biomass ash mixture. This solution is often used in construction, geology and other applications. In construction, the treatment of concrete with sodium silicate solution helps to reduce porosity in most masonry products. The increased porosity reduces water penetration. A chemical reaction takes place with the excess calcium hydroxide (Ca(OH)^2^) present in the concrete, which permanently bonds the silicates to the surface, making them much more durable and waterproof. In this study, a similar chemical reaction with calcium hydroxide from ash was expected.

**Figure 2:**
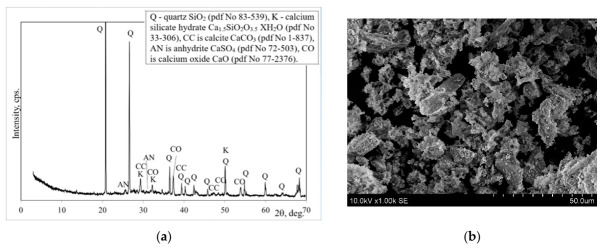
Mineral composition according to XRD (**a**) and microstructure according to SEM (**b**) of a biomass ash.

The standard sandy-pebble soil mix fr. 0/32, which is available in Lithuanian quarries (Figure 2), was used for this study; however, the physical/mechanical properties of the mix may differ from one quarry to another. The sandy-pebble soil mix 0/32 is often stored in open storage, where it can be exposed to natural atmospheric conditions, and its initial physical/mechanical properties were therefore investigated.

**Figure 3:**
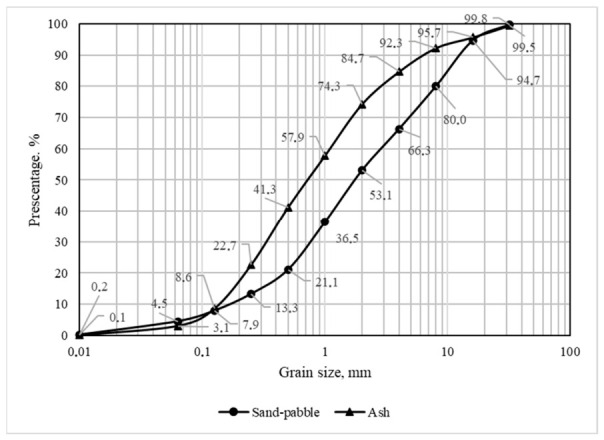
BMA and sandy-pebble soil mixture particle size distribution.

For the analysis of the granulometric composition of the materials, the samples were dried at (105 ± 5) °C to a constant weight, and the content of each fraction was determined by sieving. The results of the analyses are shown in Figure 3.

The analysis of the granulometric composition of the 0/32 sandy-pebbles fraction and the BMA revealed a fines content of 4.5% in the sandy-pebble soil mixture with a particle size of less than 0.063 mm and 3.1% in the BMA, and these materials are therefore classified as having a fines content below the 5% limit.

The properties of natural density, particle density, Proctor density, optimal moisture content and water permeability are given in Table 3.

These properties were determined for both initial soil materials, BMA and sandy pebbles. The natural density, particle density and Proctor densities of the BMA were found to be significantly lower than those of the sandy-pebble soil (see Table 3). However, the optimum moisture content of the BMA is higher and was 20% compared with the optimal moisture of sandy-pebble soil (10.5%). The water permeability coefficient of the BMA of 5.81 × 10^−5^ m/s is also lower than the water permeability coefficient of the sandy pebbles of 9.72 × 10^−5^ m/s, which indicates that soil compaction densifies the soil by forming different particle structures that affect the physical/mechanical properties of the mixtures.

#### 2.2.2. Strength Activity Index of BMA

**Figure 4:**
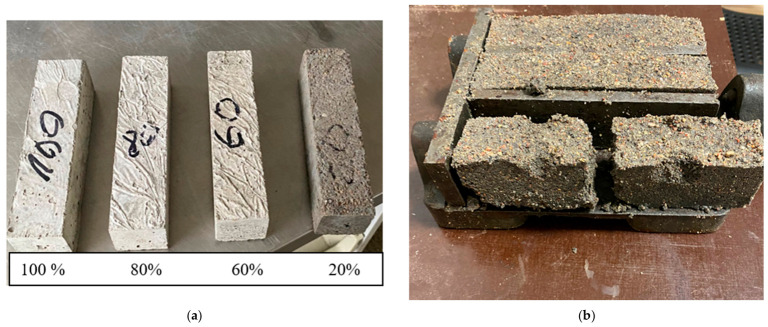
Samples were produced to assess the strength activity index (SAI) of biomass ash. Samples substituting OPC with BMA at 0, 20, 60 and 80% (**a**) and samples made of BMA only (**b**).

For the ash pozzolanic activity study (based on compressive strength according to ASTM C311) [32], it was decided to form six sample variants of nine samples each with different percentages of ash and cement. The test consisted of the typical frequently used cement CEM-I 42.5R, fine sand aggregate fr. 0/4 and biomass ash, which were relatively varied by decreasing the cement content from 0 to 100 percent, respectively (Table 4, Figure 4a).

The samples were prepared in exact proportions to ensure a comparison of the results depending on the composition of the mix. The samples formed by substituting the full 100% OPC with biomass ash (No. 7) did not set and were therefore not evaluated in the calculations (Figure 4b).

The samples with the lowest level of OPC substitution had the highest pozzolanic activity (Table 5).

The longer the hydration time, the higher the maximum strength values and the SAI as well. The lower strength values and lower SAI could be explained by the dilution effect of OPC and low-range pozzolanic reaction [33].

In accordance with EN 450-1:2012, the SAI (the ratio of the compressive strength of the mortar containing 25% BMA as OPC supplementary material to the compressive strength of the reference mortar) shall be at least 75% at 28 days. The results showed that the pozzolanic activity index of BMA was higher (0.80) than 0.75, which indicated that this ash could be used as cement supplementary materials and has pozzolanic character. Similar findings were published in other studies by Kramar et al. and Patil et al. [34,35].

## 3. Results and Discussion

### 3.1. A Complex Investigation of Sandy-Pebble and Biomass Ash Mixtures

Based on the results of the previous SAI study, the proportions of sand and BMA were determined, and six mixtures were prepared to determine the optimal Proctor density and moisture content. The studies were carried out in order to identify mixtures composed of these materials that would meet the positive performance requirements for road construction. In road construction, it is important to assess the Proctor density and water permeability of mixtures, as in many cases increasing density decreases water permeability, making waterproof materials unsuitable for use.

Proctor density and water permeability tests showed that in a mixture of sandy-pebble soil and BMA, an increasing proportion of ash decreases the density of the whole mixture from 1.96 kg/m^3^ to 1.48 Mg/m^3^, with the exception of the 80% sandy pebbles and 20% BMA. The Proctor density then increases to 2.04 Mg/m^3^, but the water permeability decreases from time to time but does not reach the threshold (≤2 × 10^−5^ m/s) where the mixture is no longer desirable for road construction (Table 5) [36]. Similar findings were reported in another study [37], which found that the application of bottom ash gradually reduces the water permeability of stabilized soils.

### 3.2. Effect of Sodium Silicate Additive on the CBR Strength of Sandy-Pebble–Biomass Ash Blends

The 20% BMA mixture exhibited the most favorable mechanical and compaction properties, including the highest Proctor density and pozzolanic activity among BMA-containing composites, and therefore served as a reference mixture with optimal baseline performance. In contrast, the 60% BMA mixture represented a high-ash, mechanically challenging composition in which the cement dilution effect was pronounced, making it suitable for assessing whether alkaline activation could enhance strength and enable the use of substantially higher BMA contents. Intermediate mixtures (40% and 80% BMA) showed monotonic transitional behavior between these two endpoints and were therefore excluded to avoid experimental redundancy while retaining engineering relevance. Soluble glass was used as an alkaline activator and was incorporated into the sandy-pebble soil and ash mixture in varying proportions, as illustrated in Table 6.

The utilization of this activator has been demonstrated to enhance the mechanical properties of the compacted mix, whilst concomitantly reducing the dustiness of the hardened mix employed in road construction [38].

Studies have shown that the Proctor density increased with increasing sodium silicate content, although the optimum moisture content of the mix was constant (see Table 6). The Proctor density of the SB/1 mixture increased from 2.04 Mg/m^3^ when only water was used in the mixture to 2.10 Mg/m^3^ when the ratio of water to sodium silicate in the mixture was 1:3. A similar trend was observed in the blend with 60% biomass ash, with an increase in Proctor density from 1.91 Mg/m^3^ to 1.96 Mg/m^3^, despite the lower initial natural density (1.32 Mg/m^3^). The Proctor density of the mixture shows a positive effect of alkali activation.

**Figure 5:**
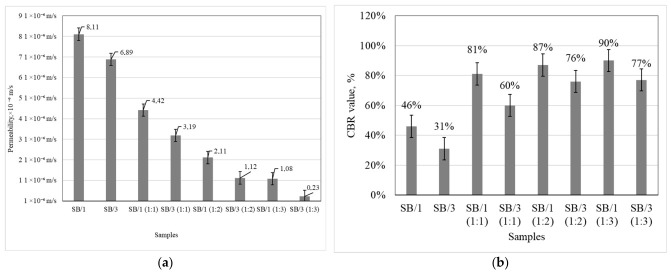
Test results for water permeability (**a**) and CBR value of sandy-pebble soil and alkali-activated biomass ash mixtures with varying amounts of soluble glass solution (**b**).

The strength of the soil was evaluated by the CBR index, which showed that the CBR values of mixtures of sandy-pebble soil (80%) and BMA (20%) increased with the soluble glass and water solution used to stabilize the mixtures (Figure 5a).

Considering the value of the CBR for the sandy pebbles (80%) and BMA (20%) with the appropriate additive content, the CBR index increased from 41% when no soluble glass solution was used in the mixture to 90% when the ratio of water to soluble glass was 1:3, but the water permeability of these mixtures decreased with increasing soluble glass amount from 8.01 × 10^−5^ m/s to 1.08 × 10^−5^ m/s when the ratio of water to soluble glass was 1:3, which does not meet the condition of ≥2 × 10^−5^ m/s.

When more BMA was included in the system, the CBR values of the mixtures for sandy-pebble soil (40%) and BMA (60%) increased, as the soluble glass stabilized the soil by forming binding chemical compounds. In this case, the CBR increased by about 31% when no soluble glass was used in the mixture and by up to 77% when the ratio of water to soluble glass was 1:3. However, the water permeability of these mixtures decreased with increasing soluble glass content in the mixture: from 6.09 × 10^−5^ m/s for a water/soluble glass ratio of 1:1 to 2.30 × 10^−6^ m/s for a water/soluble glass ratio of 1:3, which does not meet the condition of ≥2 × 10^−5^ m/s (Figure 5b).

CBR values thus depend on the BMA content of the mixtures. Higher CBR values were achieved with 20% BMA compared to CBR values achieved in mixtures with 60% BMA. Water permeability is also closely related to the amount of BMA. At the optimum BMA amount, these particles densify the sandy-pebble soil by filling voids. Along with the densifying effect, the pozzolanic properties are important. Other authors [39] confirmed these findings. Teerawattanasuk et al. [40] confirmed a positive change in CBR when stabilizing soil with an ash geopolymer. In this case, the soil was densified, which had a positive effect on the change in CBR.

It should be noted that pure biomass ash alone does not exhibit condensation or strength development due to the lack of sufficient reactive alkalinity and binding mechanisms. In contrast, when biomass ash is combined with sandy-pebble soil and activated with sodium silicate, a mechanically stable granular skeleton is formed, and soluble alkalis initiate pozzolanic and geopolymeric reactions. This alkaline activation leads to gel formation, particle bonding, and structural condensation of the mixture. A comparison with non-activated (water-only) biomass ash–gravel mixtures indicates that physical compaction alone is insufficient to achieve comparable consolidation, highlighting the crucial role of alkali excitation in strength development.

### 3.3. Mineral Composition Based on XRD and Infrared Spectroscopy Analyses of Alkali-Activated Sandy–Pebble Soil and Biomass Bottom Ash

**Figure 6:**
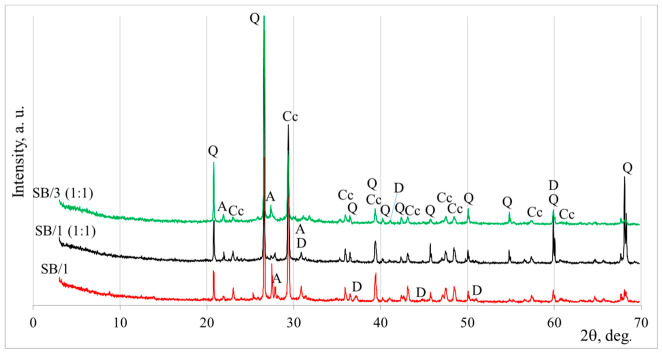
XRD curves of alkali-activated sandy-pebble soil and biomass bottom ash blends. Notes: A is anorthoclase (Na_0.667_ K_0.333_) (AlSi_3_O_8_), PDF No. 75-1630; Cc is calcite (CaCO_3_), PDF No. 83-578; Q is quartz (SiO_2_), PDF No. 77-1060; and D is dolomite (CaMgCO_3_), PDF No. 74-1687.

The mineral composition of the alkali-activated sandy–pebble soil and biomass bottom ash (BMA) blends was investigated by X-ray diffraction (XRD), and the obtained diffraction patterns are presented in Figure 6. The XRD results indicate that the crystalline phases present in the alkali-activated systems are mainly inherited from the raw materials, while no new well-crystalline reaction products were clearly detected after activation.

The dominant crystalline phase identified in all mixtures is quartz (Q, SiO_2_; PDF No. 77-1060), originating primarily from the sandy-pebble soil and BMA. Calcite (C, CaCO_3_; PDF No. 83-578) was detected mainly in the mixtures containing higher amounts of BMA, whereas dolomite (D, CaMg(CO_3_)_2_; PDF No. 74-1687) was predominantly observed in mixtures with higher sandy-pebble soil contents.

Anorthoclase (A, (Na_0.667_ K_0.333_) (AlSi_3_O_8_); PDF No. 75-1630) was identified as a minor aluminosilicate phase in the sandy-pebble soil. This phase may act as a potential source of alumina and silica for the alkali-activation process. Under alkaline conditions, partial dissolution of anorthoclase cannot be excluded and could contribute to the formation of amorphous aluminosilicate hydration products.

**Figure 7:**
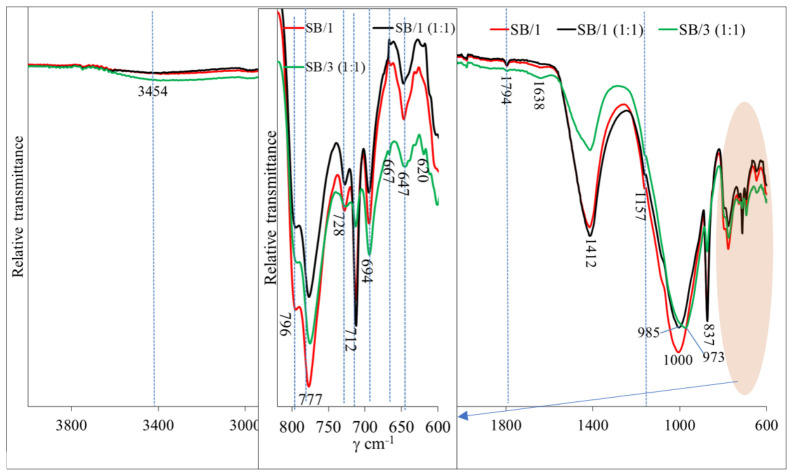
FT-IR spectra of alkali-activated sandy-pebble soil and biomass bottom ash blends.

Overall, the XRD analysis indicates that alkali activation is primarily associated with the development of an amorphous phase, while crystalline minerals such as quartz, anorthoclase, calcite, and dolomite are largely retained in the system; therefore, FT-IR spectroscopy was further employed to evaluate the hydration products formed after alkaline activation (Figure 7).

OH and HOH vibrations. The weak bands at 3454 and 1638 cm^−1^ are attributed to the stretching vibration of OH and bending vibration of HOH in C-S/A-H gels [41,42]. The highest intensity of both these bands was found in the SB/3 (1:1) sample due to the more reactive mixture of initial materials when a higher amount of BMA is substituted for natural sand. In this case, the higher number of C-S/A-H gels may have been formed by the alkali activation with soluble glass.

Quartz peaks. Quartz is detected according to the bands at 777 cm^−1^, 796 cm^−1^ (double peak) and 694 cm^−1^ [35]. The intensity of these bands gradually decreased with increasing BMA content in the initial material mixtures and with the use of an alkaline activator.

Carbonate vibrations. The strong bands at 1412 cm^−1^ and the sharp bands at 837 and 712 cm^−1^ are attributed to CO_3_ (CaCO_3_ and Na_2_CO_3_) vibrations [43,44]. Similar to quartz, increasing the amount of BMA in the system (SB/3 (1:1) sample) and adding soluble glass resulted in a decrease in carbonate-related bands. It is possible that calcium and sodium compounds were incorporated into the newly formed hydration products.

Main geopolymerization band. The main and strongest peaks are in the range of 973–1000 cm^−1^ and were assigned to asymmetric stretching vibrations of Si-O-Si(Al). Mathivet et al. [45] found that this broad band is attributed to the formation of an N-A-S-H gel in the geopolymer matrix. When BMA was included in the mixtures, this peak shifted to the side with lower wavenumbers. A similar main peak shift was detected by Valcke et al. [46], who explained that it could be related to the formation of geopolymerization products with higher amounts of aluminum. The highest peak was detected for the SB/1 sample, and it could be related to the amount of quartz from the sand in the system. Similar findings were published by Liu et al. [47], and they found that the bands at about 973–1000 cm^−1^ could overlap with the bands that are attributed to vibrations of Si-O (quartz). Another reason could be the higher amount of calcium in the system, which led to the formation of more crystalline hydration products and for that reason decreasing the amount of gel and decreasing the main broad bands at 973 and 985 cm^−1^ [48]. These newly formed amorphous and crystalline hydrates positively affected the higher mechanical properties such as the SB/3 (1:1) sample.

### 3.4. Microstructural Investigation of Alkali-Activated Sandy Pebbles and Biomass Bottom Ash

**Figure 8:**
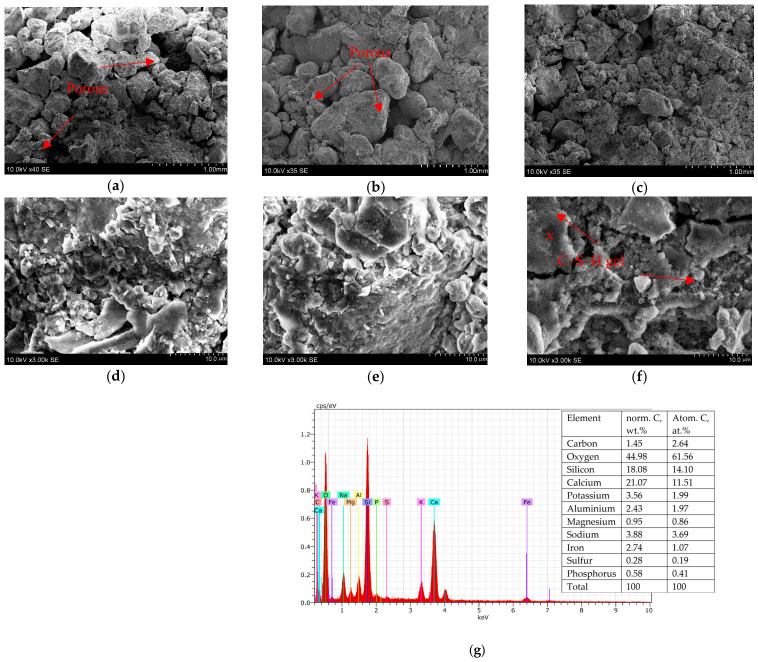
SEM pictures of sandy-pebble and biomass bottom ash blends at different magnifications: (**a**,**d**) are SB/1, (**b**,**e**) are SB/1 (1:1), and (**c**,**f**) are SB/3 (1:1). Chemical characterization by EDS of the fibrous reaction product is marked as x in the graphic (**g**).

Three samples were chosen for the evaluation of microstructure, SB/1, SB/1 (1:1) and SB/3 (1:1), according to Table 6. Samples made of 80% sandy pebbles and 20% BMA had a more open structure, and the stabilized soil was slightly porous (Figure 8a,b) compared to samples with a higher amount of BMA (Figure 8c). Because BMA is finer by nature, increasing the BMA in the mixtures led to more compact microstructures with less porosity.

The effect of soluble glass resulted in a decrease in the heterogeneity of the SB/1 (1:1) and f SB/3 (1:1) samples (Figure 8e,f) compared to the SB/1 sample (Figure 8d). In this case, a calcium silicate hydrate (C-S-H gel) morphology with a fibrous form can be detected, especially in the sample with a higher BMA content (Figure 8f). A similar microstructure of the C-S-H gel was found by Velardo et al. [49]. Thus, the microstructure of the BMA/soluble glass-stabilized sandy-pebble soil had a significant influence on the main geotechnical properties. The EDS spectrum (Figure 8g) confirms the presence of this fibrous C–S–H reaction product, supporting the microstructural observations and aligning with findings reported in similar alkali-activated systems [50]. Overall, the microstructural evidence confirms that the synergistic effect of fine BMA particles and soluble glass activation promotes the formation of dense C–S–H gel networks, which in turn govern the improved strength, stiffness, and durability of the stabilized sandy-pebble soils.

As demonstrated, mixtures exhibiting the most compact gel-rich microstructures—particularly SB/1 (1:1) and SB/3 (1:1)—also achieved the highest increases in Proctor density (up to +0.06–0.09 Mg/m^3^) and CBR strength (up to +49 percentage points for 20% BMA and +46 percentage points for 60% BMA). These improvements coincide with the formation of continuous C–S–H networks observed in SEM and confirmed by FT-IR and EDS analyses. The strong alignment between microstructural indicators (reduced pore connectivity, fibrous gel morphology) and engineering performance metrics (strength, stiffness, permeability) provides a mechanistic basis for predicting mixture behavior. Such quantifiable structure–property relationships are essential for the practical implementation of alkali-activated BMA–sand systems in road construction, enabling more accurate mixture design and performance optimization under field conditions.

This study also highlights the broader practical relevance of using biomass bottom ash as a sustainable stabilizing component. The incorporation of BMA not only enhances the engineering performance of sandy-pebble soils but also contributes to resource efficiency by utilizing a significant industrial by-product. The alkali-activated mixtures demonstrated improved structural integrity and load-bearing capacity, indicating their suitability for road foundation layers and similar geotechnical applications. By integrating waste valorization with performance-driven material design, the approach offers a viable alternative to conventional stabilizers and supports the development of more environmentally responsible construction practices.

It should be noted that curing at ambient temperature may restrict the full progression of alkali-activated reactions, implying that the mechanical improvements observed in this study likely represent a conservative estimate of the material’s performance. Consequently, future research should incorporate comparative elevated-temperature curing experiments to more thoroughly evaluate the activation efficiency of soluble glass and its interaction with low-reactivity biomass ash.

## 4. Conclusions

The findings of this study indicate that BMA has the potential to act as a substitute for sandy-pebble soil in geotechnical applications. According to the strength activity index, BMA has a pozzolanic nature, and it is higher than 0.75. The leaching analysis of hazardous compounds in BMA showed that the hazardous chemical elements do not exceed the limits for heavy metals. The soluble glass, which was used as an alkali activator, promotes the chemical reaction between active compounds of ash by forming a binding matrix. These newly formed binding compounds, such as amorphous and crystalline hydrates, create a stabilizing effect on sandy-pebble soil. The microstructure improved after soil stabilization with BMA and soluble glass, which formed binding compounds such as N-A-S-H and C-S-H gels. These results were confirmed by FT-IR and SEM analysis. The study of the dynamics of the variation in the Proctor density and water permeability shows that an increase in BMA in sandy-pebble soil decreases the density of the mixture from 1.96 Mg/m^3^ to 1.48 Mg/m^3^. When 20% of BMA was included in the sandy-pebble soil, the Proctor density increased to 2.04 Mg/m^3^, but the water permeability decreased with increasing BMA content from 8.11 × 10^−5^ m/s to 5.91 × 10^−5^ m/s but does not reach the threshold (≤2 × 10^−5^ m/s). Higher CBR values were found for the SB/1 samples (with and without alkali activation) compared to the SB/3 samples with a higher amount of BMA. This phenomenon can be explained through the findings of BMA pozzolanic properties. The mechanical properties of the soil are improved by a combination of geopolymerization reactions and the granulometric composition. In all investigated cases, geopolymerization reactions increased CBR values.

## Figures and Tables

**Table 1:** Chemical composition according to X-ray dispersive fluorescence analysis (XRF), %.

Oxide	CaO	SiO_2_	Al_2_O_3_	Fe_2_O_3_	MgO	K_2_O	Na_2_O	SO_3_	CI	TiO_2_	SrO	P_2_O_5_	MnO	BaO	ZnO	LOI
OPC	63.02	19.51	4.26	3.20	3.3	1.0	0.11	3.30	0.03	0.17		-	-	-	-	2.1
BMA	13.6	72.3	3.79	0.85	2.12	4.08	0.53	0.31	0.04	0.09	0.02	1.90	0.23	0.09	0.07	-

**Table 2:** Leaching of heavy metals from the biomass ash determined according to flame atomic absorption spectrophotometry (mg/L).

Elements	Cd	Ni	Pb	V	Cr	Zn
mg/L	0.00058	0.0028	0.0882	0.142	0.0047	0.0107

**Table 3:** Biomass ash and sandy-pebbles mixture density, water permeability and optimal moisture.

Samples	Natural Density, Mg/cm^3^	Particles Density, Mg/cm^3^	Proctor Density, Mg/cm^3^	Optimal Moisture, %	Water Permeability, m/s
Biomass ash	1.10	2.12	1.48	20.0	5.81 × 10^−5^
Sandy pebbles	1.58	2.67	1.96	10.5	9.72 × 10^−5^

**Table 4:** Composition of initial materials for the mortars, with BMA and evaluation of pozzolanic activity.

No.	OPC, %	BMA, %	Sand fr. 0/4, g	Water, mL	Compressive Strength, MPa		Pozzolanic Activity Index	
					After 7 Days	After 28 Days	After 7 Days	After 28 Days
1	100	0	1350	225	34.47	41.3	1	1
2	80	20	1350	225	23.26	36.93	0.67	0.89
3	75	25	1350	225	20.16	33.12	0.59	0.80
4	60	40	1350	225	12.97	20.96	0.37	0.51
5	40	60	1350	260	6.25	10.45	0.18	0.25
6	20	80	1350	275	0.88	1.22	0.03	0.03
7	0	100	1350	290	0	0	0	0

**Table 5:** Results of Proctor density and optimum moisture content studies for sandy-pebble soil and BMA mixtures.

Samples	Sandy Pebbles, %	Biomass Ash, %	Natural Density, Mg/m^3^	Proctor Density, Mg/m^3^	Optimum Moisture, %	Permeability, m/s
S	100	0	1.58	1.95	10.5	9.72 × 10^−5^
SB/1	80	20	1.61	2.04	11.2	8.11 × 10^−5^
SB/2	60	40	1.43	1.94	12.6	8.01 × 10^−5^
SB/3	40	60	1.32	1.91	13.4	6.89 × 10^−5^
SB/4	20	80	1.28	1.68	16.7	5.91 × 10^−5^
B	0	100	1.12	1.48	20.0	5.81 × 10^−5^

**Table 6:** The mixed proportions of sandy pebbles and alkali-activated biomass ash.

Samples	Sandy Pebbles, %	Biomass Ash, %	Natural Density, Mg/m^3^	Weight Ratio of Water to Soluble Glass	Proctor Density, Mg/m^3^	Optimum Moisture, %
SB/1	80	20	1.61	1:0	2.04	11.2
SB/1 (1:1)	80	20	1.61	1:1	2.06	11.2
SB/1 (1:2)	80	20	1.61	1:2	2.08	11.2
SB/1 (1:3)	80	20	1.61	1:3	2.10	11.2
SB/3	40	60	1.32	1:0	1.91	13.4
SB/3 (1:1)	40	60	1.32	1:1	1.93	13.4
SB/3 (1:2)	40	60	1.32	1:2	1.95	13.4
SB/3 (1:3)	40	60	1.32	1:3	1.96	13.4

## Data Availability

The original contributions presented in this study are included in the article. Further inquiries can be directed to the corresponding author.
